# Inhibition of TDP-43 Accumulation by *Bis*(thiosemicarbazonato)-Copper Complexes

**DOI:** 10.1371/journal.pone.0042277

**Published:** 2012-08-03

**Authors:** Sarah J. Parker, Jodi Meyerowitz, Janine L. James, Jeffrey R. Liddell, Takashi Nonaka, Masato Hasegawa, Katja M. Kanninen, SinChun Lim, Brett M. Paterson, Paul S. Donnelly, Peter J. Crouch, Anthony R. White

**Affiliations:** 1 Department of Pathology, The University of Melbourne, Parkville, Victoria, Australia; 2 Department of Neuropathology and Cell Biology, Tokyo Metropolitan Institute of Medical Science, Setagaya-ku, Tokyo, Japan; 3 Bio21 Molecular Science and Biotechnology Institute, Parkville, Victoria, Australia; 4 School of Chemistry, The University of Melbourne, Parkville, Victoria, Australia; 5 Mental Health Research Institute, Parkville, Victoria, Australia; Hertie Institute for Clinical Brain Research and German Center for Neurodegenerative Diseases, Germany

## Abstract

Amyotrophic lateral sclerosis (ALS) is a progressive, fatal, motor neuron disease with no effective long-term treatment options. Recently, TDP-43 has been identified as a key protein in the pathogenesis of some cases of ALS. Although the role of TDP-43 in motor neuron degeneration is not yet known, TDP-43 has been shown to accumulate in RNA stress granules (SGs) in cell models and in spinal cord tissue from ALS patients. The SG association may be an early pathological change to TDP-43 metabolism and as such a potential target for therapeutic intervention. Accumulation of TDP-43 in SGs induced by inhibition of mitochondrial activity can be inhibited by modulation of cellular kinase activity. We have also found that treatment of cells and animal models of neurodegeneration, including an ALS model, with bioavailable *bis*(thiosemicarbazonato)copper^II^ complexes (Cu^II^(btsc)s) can modulate kinase activity and induce neuroprotective effects. In this study we examined the effect of diacetylbis(-methylthiosemicarbazonato)copper^II^ (Cu^II^(atsm)) and glyoxalbis(-methylthiosemicarbazonato)copper^II^ (Cu^II^(gtsm)) on TDP-43-positive SGs induced in SH-SY5Y cells in culture. We found that the Cu^II^(btsc)s blocked formation of TDP-43-and human antigen R (HuR)-positive SGs induced by paraquat. The Cu^II^(btsc)s protected neurons from paraquat-mediated cell death. These effects were associated with inhibition of ERK phosphorylation. Co-treatment of cultures with either Cu^II^(atsm) or an ERK inhibitor, PD98059 both prevented ERK activation and blocked formation of TDP-43-and HuR-positive SGs. Cu^II^(atsm) treatment or ERK inhibition also prevented abnormal ubiquitin accumulation in paraquat-treated cells suggesting a link between prolonged ERK activation and abnormal ubiquitin metabolism in paraquat stress and inhibition by Cu. Moreover, Cu^II^(atsm) reduced accumulation of C-terminal (219–414) TDP-43 in transfected SH-SY5Y cells. These results demonstrate that Cu^II^(btsc) complexes could potentially be developed as a neuroprotective agent to modulate neuronal kinase function and inhibit TDP-43 aggregation. Further studies in TDP-43 animal models are warranted.

## Introduction

Amyotrophic lateral sclerosis (ALS) is a fatal adult-onset motor neuron disease in which death of upper and lower motor neurons leads to progressive brain and spinal cord deterioration. Patients with ALS rarely survive more than a few years after diagnosis with respiratory failure the most common cause of death [1]. Early studies into ALS identified familial mutations in a small percentage of patients. The most well-characterized of these has been mutations to superoxide dismutase 1 (SOD1). Extensive cell and animal-based studies have focused on SOD1 biology in ALS [2], [3], however, these mutations only account for around 20% of familial ALS cases and approximately 2–3% of all ALS patients [4], [5].

In 2006, TAR DNA binding protein 43 (TDP-43) was identified as the major protein constituent of ubiquitinated neuronal inclusions in non-SOD1 ALS cases [6], [7] as well as in cases of frontotemporal dementia (FTD) with ubiquitin inclusions. ALS and FTD cases involving TDP-43 pathological changes are now referred to collectively as primary TDP-43 proteinopathies [8]. Subsequently, TDP-43-positive inclusions have been identified in a number of neurodegenerative diseases. In these cases, the TDP-43 identification is referred to as a secondary TDP-43 proteinopathy [8]. While the role of abnormal TDP-43 accumulation in both primary and secondary TDP-43 proteinopathies is not yet fully understood, the identification of TDP-43 mutations associated with ALS and FTD has provided clear evidence that altered TDP-43 processing can be a primary cause of neurodegeneration and is not just a secondary phenomenon [9], [10].

TDP-43 is a 414 amino acid protein of the heterogeneous nuclear ribonucleoprotein (hnRNP) family and consists of two RNA recognition motifs and a C-terminal glycine rich region [8], [11]. It has a number of reported roles including transcription, pre-mRNA splicing, and transport and stabilization of mRNA [8]. TDP-43 contains two caspase 3 consensus cleavage sites, which result in formation of C-terminal fragments (CTFs) of 35 kDa and 25 kDa that accumulate in the cytosol [8]. The majority of TDP-43 mutations occur in the C-terminal region and CTFs are commonly identified in ALS and FTD inclusions.

The hallmark neuropathological features of primary TDP-43 proteinopathies include clearing of TDP-43 from the nucleus and accumulation of TDP-43 in cytoplasmic inclusions. These inclusions are enriched in ubiquitinated and hyperphosphorylated (phospho-Ser409/410) CTF-TDP-43 [8], [11]. Recent cell studies have shown that transfection with CTF-TDP-43 can accurately re-capitulate the histopathological findings of ALS and FTD with accumulation of cytosolic ubiquitinated CTF-TDP-43 aggregates [12]–[14]. In addition, transfection with these constructs can result in neurotoxicity and cell death although the pathways involved are not known [13].

**Figure 1:**
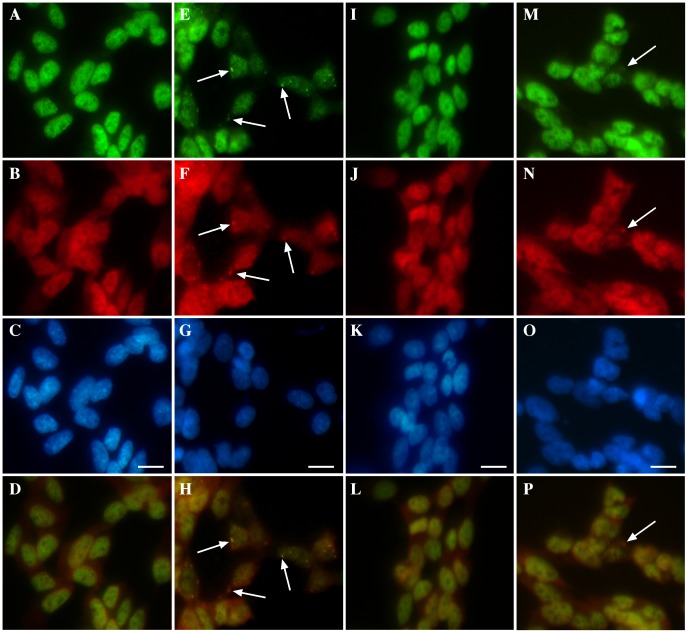
Effect of Cu^II^(atsm) and Cu^II^(gtsm) on TDP-43 and HuR localization in SY5Y cells. Cells were exposed overnight with 1 mM paraquat in the presence or absence of 1 µM Cu^II^(atsm) or 50 nM Cu^II^(gtsm) and TDP-43 and HuR localization was examined by immunofluorescence. **A–D**: untreated, **E–H**: paraquat, **I–L**: paraquat and Cu^II^(atsm), **M–P**: paraquat and Cu^II^(gtsm). Green  =  TDP-43, red  =  HuR, blue  =  DAPI. Bottom panels  =  merged images of TDP-43 and HuR above. Arrows indicate TDP-43 and HuR SGs. Bar  = 10 µm.

The early disease processes associated with abnormal TDP-43 metabolism are not well understood. Recent studies have shown that an early change to TDP-43 processing and metabolism in ALS may involve its localization to RNA stress granules (SGs) [15], [16]. SGs are cytoplasmic sites of stalled mRNA pre-initiation complexes induced by various cellular stresses. Upon stress induction, the cell stalls mRNA translation of non-critical proteins to shift energy expenditure to key repair and survival proteins [17]. During stress, TDP-43 is recruited to SGs in a variety of cells [15], [16], [18], [19]. This localization appears to depend on residues 216–315 in the C-terminal region and the first RNA recognition motif [18]. Importantly, studies have now shown that TDP-43 can be localized with cytosolic SG markers in ALS spinal cord tissue [16]. Moreover, FUS, another hnRNP protein associated with ALS, has also been identified in ALS SGs [20], [21].

While these studies have begun to delineate some of the initial processes in TDP-43 accumulation, it is not known whether this early stage of TDP-43 aggregation can be developed into a therapeutic target. Recently, we developed a cell model involving paraquat-induced mitochondrial inhibition in neurons and demonstrated that this stress induced changes to TDP-43 that re-capitulated *in vivo* hallmarks of disease [22]. These included loss of TDP-43 from the nucleus, accumulation of TDP-43 in the cytosol, formation of a TDP-43 C-terminal fragment, and aggregation of TDP-43 into cytosolic SGs, a portion of which were ubiquitinated. Moreover, accumulation of TDP-43 in the SGs was mediated through activation of the stress kinase c-Jun N-terminal kinase (JNK) [22]. In addition, inhibition of extracellular signal-regulated kinase (ERK) could prevent formation of TDP-43-and human antigen R- (HuR)-positive SGs induced by paraquat [22]. These studies clearly established a potentially important role for stress-associated kinase activation in early TDP-43 aggregation.

In the present study, we have used this cell model to examine the effect of bis(thiosemicarbazonato)-copper^II^ complexes (Cu^II^(btsc)s) on TDP-43 SG formation. These complexes have traditionally been investigated for their potential as imaging agents for hypoxic tumors [23]. However, recent studies have demonstrated that Cu^II^(btsc)s have a potent neuroprotective effect in several different mouse models of neurodegenerative disorders, including models of Alzheimer’s disease, Parkinson’s disease and ALS [24]–[26]. While the mechanism is not fully understood, the neuroprotective action of Cu^II^(btsc)s is likely related to their ability to modulate kinase signaling mechanisms including Akt, ERK and JNK in both cell culture and animal models [24], [27]–[29]. In a recent study, we have shown that diacetyl*bis*(methylthiosemicarbazonato)-copper^II^ (Cu^II^(atsm)) has a potent protective effect in an animal model of ALS (SOD1G93A) [26]. Interestingly, Cu^II^(atsm) also modulated TDP-43 processing in the SOD1G93A murine model. Treatment of SOD1G93A mice with Cu^II^(atsm) inhibited accumulation of phosphorylated TDP-43 in the spinal cord of these mice [26]. Based on these and our previously published studies showing kinase-dependent neuroprotective action of Cu^II^(btsc)s and other Cu-complexes in animal and cell models [29], the present study examined if Cu^II^(btsc)s could modify the processing and localization of TDP-43 to SGs. We found that Cu^II^(atsm) and glyoxalbis(methylthiosemicarbazonato)-copper^II^ (Cu^II^(gtsm)) were potent inhibitors of SG formation, blocking accumulation of TDP-43 and HuR into SGs caused by treatment with paraquat. Further studies showed that the protective action was associated with inhibition of stress-induced ERK phosphorylation by Cu^II^(atsm). The protective effect of Cu^II^(atsm) on TDP-43 aggregation was also associated with an inhibitory effect on accumulation of ubiquitinated proteins and decreased paraquat neurotoxicity. Moreover, Cu^II^(atsm) reduced accumulation of C-terminal (219–414) TDP-43 in transfected SH-SY5Y cells. These studies show for the first time that treatment of neuronal-like cells with a copper-complex can inhibit stress-induced cell toxicity and prevent early aggregation of TDP-43 into SGs. This may offer a potential insight into novel targets and therapeutic treatments for TDP-43 proteinopathies.

## Methods

### Materials

4′,6′ Diamino-2-phenylindole dihydrochloride (DAPI) was obtained from Invitrogen (Mount Waverley, Victoria, Australia). N,N′-Dimethyl-4,4′-bipyridinium dichloride (paraquat), rotenone, 1-methyl-4-phenylpyridinium (MPP+) and sodium arsenite, were from Sigma Aldrich (Sydney, NSW, Australia) and LDH assay kit was purchased from Roche Diagnostics (Castle Hill, NSW, Australia). SP600125 and PD98095 were purchased from Merck Biosciences (Melbourne, Victoria, Australia).

Polyclonal TDP-43 antisera were purchased from Proteintech Group (Chicago, IL, USA). Antisera to ubiquitin were from Santa Cruz Biotechnology (Santa Cruz, CA, USA). Monoclonal antisera to HuR were obtained from Invitrogen (Mount Waverley, Victoria, Australia). Antisera to total and phosphorylated forms of ERK and JNK, as well as antibodies to actin and GAPDH were purchased from Cell Signalling Technologies (Arundel, Queensland).

**Figure 2:**
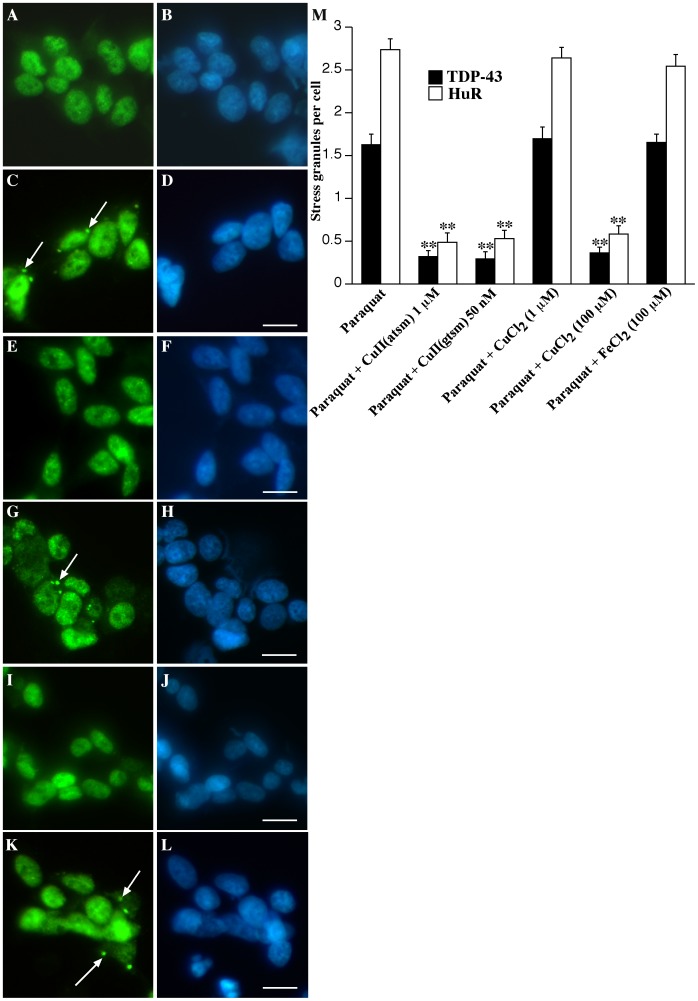
Effect of Cu^II^(btsc)s and metals on TDP-43 and HuR SGs in SY5Y cells. Cells were treated overnight with 1 mM paraquat in the presence or absence of 1 µM Cu^II^(atsm), 1 and 100 µM CuCl_2_ and 100 µM FeCl_2_. **A–B**: untreated, **C–D**: paraquat, **E–F**: paraquat and Cu^II^(atsm), **G–H**: paraquat and 1 µM CuCl_2_, **I–J**: paraquat and 100 µM CuCl_2_, **K–L**: paraquat and 100 µM FeCl_2_. Green  =  TDP-43, blue  =  DAPI. Arrows indicate SGs. Bar  = 10 µm. **M**: SGs (TDP-43 or HuR-positive) per cell after overnight treatment with 1 mM paraquat and co-treatment with Cu^II^(atsm), Cu^II^(gtsm), CuCl_2_ (1 and 100 µM) or FeCl_2_. **p<0.01 compared to paraquat treatment.

### Cell Culture

The cell lines used in this study were the human neuroblastoma SH-SY5Y cell line and human epithelial HeLa cell line. Cells were passaged and maintained in DMEM plus 5% FBS (HeLa cells) or DMEM/F12 plus 10% FBS (SH-SY5Y cells) as previously reported [22]. All cells were grown in 5% CO_2_ at 37°C.

### Cell Lysis Assay

An assay for cell death (LDH) was performed as previously described [30].

### Bis(thiosemicarbazonato)-copper^II^ Complexes

Cu^II^(btsc)s were synthesized as previously described [27], [31]–[34]. Cu^II^(btsc)s were prepared as 10 mM stock solutions in DMSO and added at indicated concentrations. CuCl_2_ and FeCl_2_ 10 mM stock solutions were prepared in dH_2_O and added at indicated concentrations.

### Exposure of Cells to Stress

Cells were grown in 24 or 6-well plates or on 15 mm coverslips (for immunofluorescence) for 2–3 days before experiments (∼80% confluent). Paraquat, rotenone, MPP+ or sodium arsenite were prepared in dH_2_O and added at indicated concentrations and the medium was briefly mixed by aspiration. Incubations were performed for periods stated in individual experiments. Where indicated, cells were co-treated with kinase inhibitors (SP600125 (JNK) or PD98095 (ERK)) from stock solutions prepared at 10 mM in DMSO. Control cultures were treated with vehicle alone. For immunoblotting, cells were harvested into Phosphosafe Extraction Buffer (Merck Biosciences, San Diego, CA, USA) containing protease inhibitor cocktail (Roche Diagnostics) and stored at −80°C until use. For immunofluorescence studies, cells were grown on glass coverslips and fixed after treatment in 4% w/v paraformaldehyde for 30 min.

**Figure 3:**
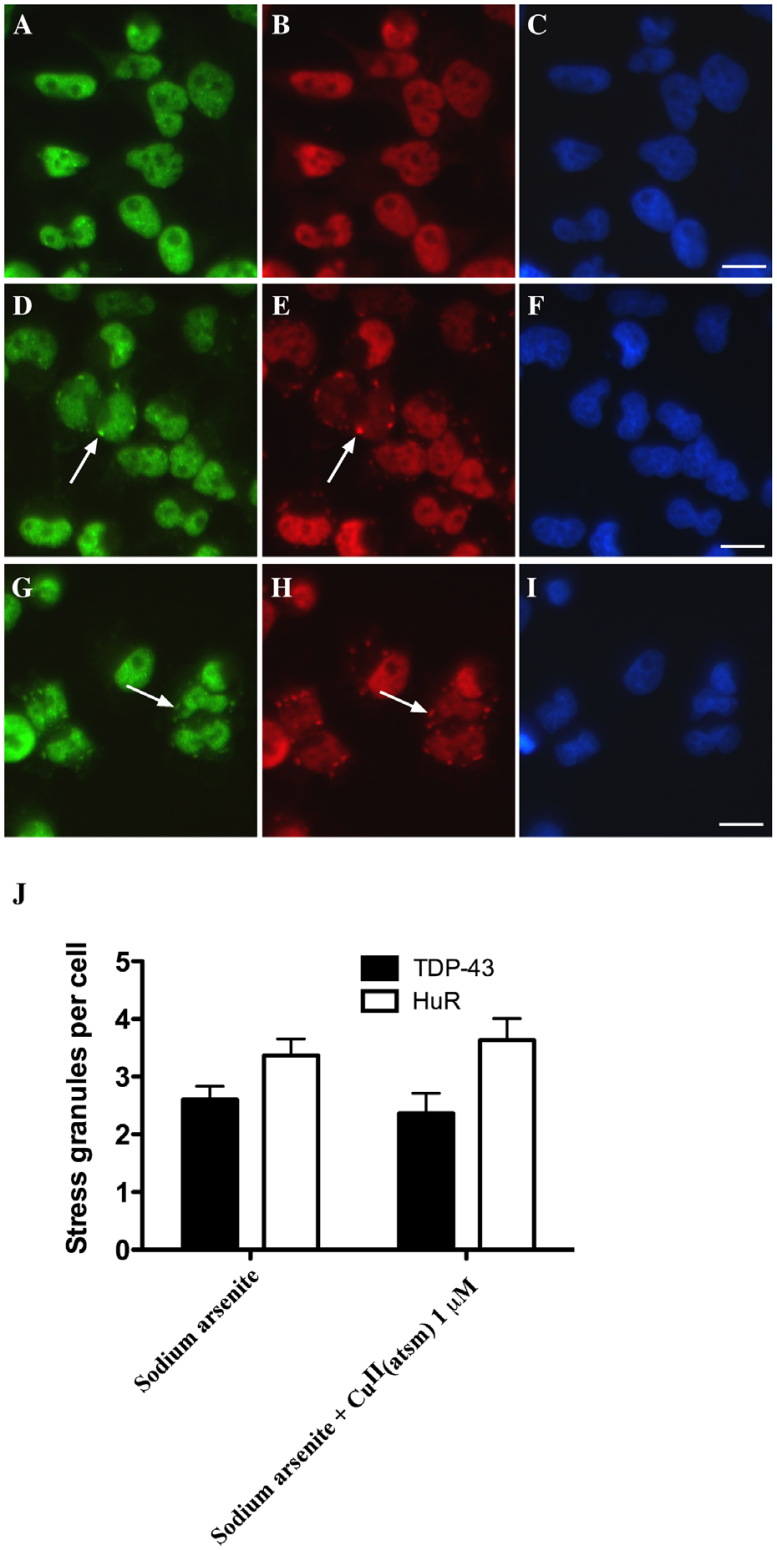
Effect of Cu^II^(atsm) on SGs in HeLa cells induced by sodium arsenite. Cells were treated for 1 hr with 500 µM sodium arsenite in the presence or absence of 1 µM Cu^II^(atsm) (1 hr) and TDP-43 and HuR were examined by immunofluoresence. **A–C**: untreated, **D–F**: sodium arsenite treated, **G–I**: sodium arsenite and Cu^II^(atsm). Green  =  TDP-43, red  =  HuR, blue  =  DAPI. Arrows indicate TDP-43 and HuR SGs. Bar  = 10 µm. **J**: SGs (TDP-43 or HuR-positive) per cell after 1 hr treatment with 500 µM sodium arsenite and co-treatment with 1 µM Cu^II^(atsm).

### ATP Assay

Cell lysates were analyzed for ATP content using an ATP Assay kit (Sigma-Aldrich) as previously reported [35].

### In-gel Superoxide Dismutase (SOD) Assay

Cells were grown and treated in 6-well (9.62 cm^2)^ multiwell plates (Nunc, Rochester, NY). After treatment with paraquat and or Cu^II^(btsc)s or CuCl_2_, cell samples were collected and centrifuged at 720 g for 10 min. Supernatant was removed and the cell suspension was re-suspended in 60 µl Phosphosafe Extraction Buffer with 5% (v/v) DNAse Sigma Aldrich (Sydney, NSW, Australia) and 1% phenylmethylsulfonyl fluoride (v/v). The suspension was then centrifuged at 15,000 g and the supernatant was collected and normalized to 8 µg/µl using the BCA assay. The cell suspension was then diluted 1∶2 in sample buffer (0.5 M Tris, pH 6.8, 50% glycerol and 1% (w/v) bromophenol blue in dH_2_O). 10 µl was loaded per well of non-denaturing polyacrylamide gels without SDS. Gels were run in Tris-glycine running buffer at 150 V for 1 hr. Gels were incubated in the dark in nitrotetrazolium blue chloride (Sigma Aldrich) solution (2 mg per ml dH_2_O) for 20 min on a rocking platform in the dark, at room temperature. Nitrotetrazolium blue chloride solution was removed from gels and replaced with developer solution (9.8% potassium orthophosphate, 0.02% riboflavin, and 8.48% TEMED. Gels were incubated for 15 min on a rocking platform in the dark, at room temperature. After the incubation, gels were illuminated (natural light) for 1 hr. Gels were subsequently imaged using a Fujifilm LAS3000 Imager (Berthold, Bundoora, Australia). Quantification of SOD activity was done using Image J analysis, comparing levels of SOD activity based on nitrotetrazolium blue reduction.

### Western blot Analysis of Protein Expression and Phosphorylation

Cell lysates prepared in Phosphosafe lysis buffer were mixed with electrophoresis SDS sample buffer and separated on 12% SDS-PAGE Tris-glycine gels. Proteins were transferred to PVDF membranes and blocked with 4% skim milk solution in PBST before immunoblotting for total or phospho-specific proteins. For detection of total TDP-43, membranes were probed with polyclonal antisera (1∶1,500) against TDP-43. For detection of total and phospho-forms of JNK and ERK, membranes were probed with anti-JNK or anti-ERK (each at 1∶5,000) and antisera to phospho-forms of each protein (each at 1∶5,000). Secondary antiserum was rabbit-HRP at 1∶5,000 dilution. Blots were developed using GE Healthcare ECL Advance Chemiluminescence and imaged on a Fujifilm LAS3000 imager. Expression of GAPDH or actin was determined using antisera at 1∶5,000 and 1∶3,000 respectively for protein loading controls where necessary.

**Figure 4:**
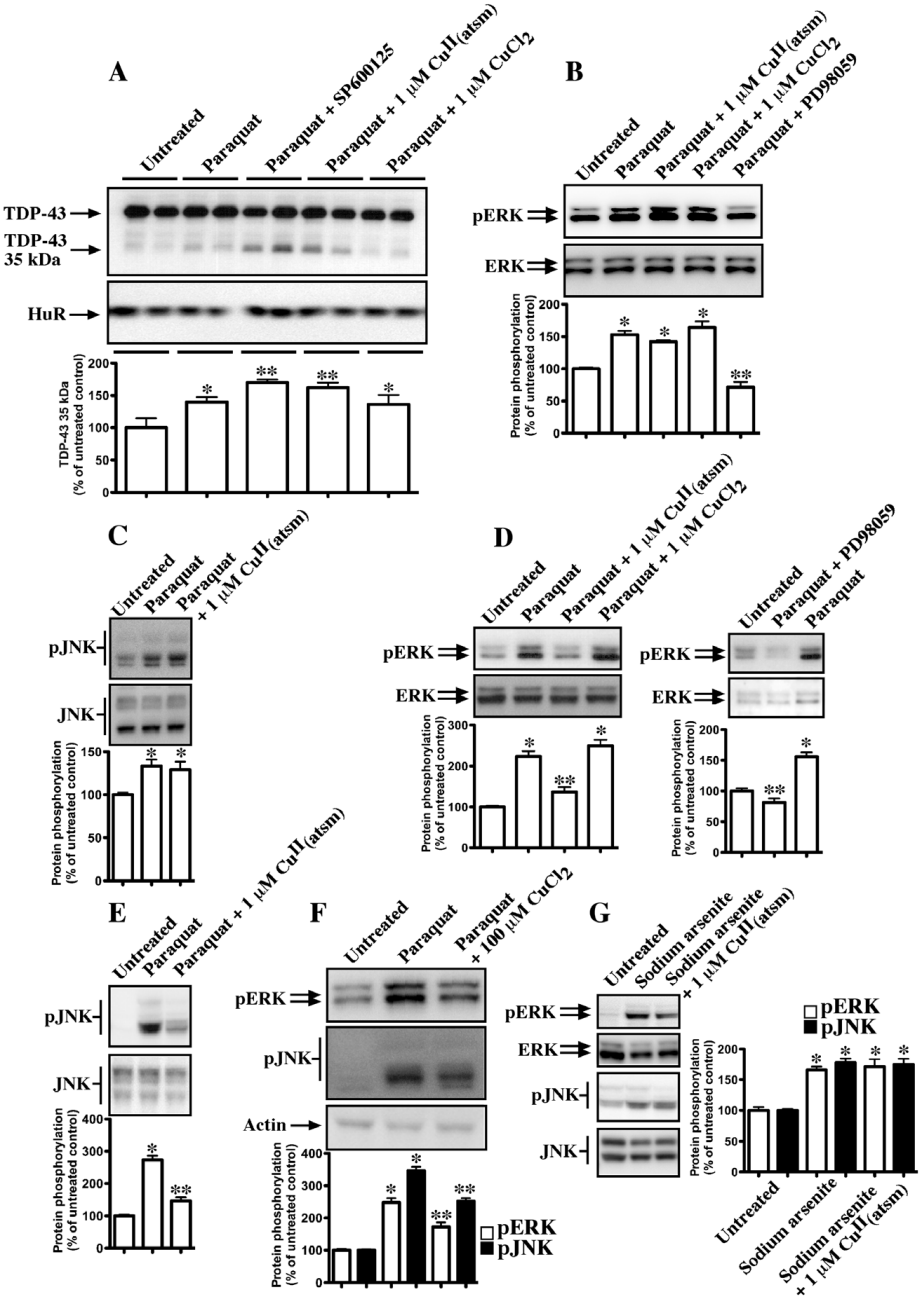
Effect of Cu^II^(atsm) on TDP-43, HuR and kinase phosphorylation. Graphs show densitometric analysis of altered protein expression or phosphorylation from two-three separate experiments relative to loading controls. **A**: Cells were treated with 1 mM paraquat overnight in the presence or absence of 20 µM SP600125, 1 µM Cu^II^(atsm) or 1 µM CuCl_2_ and immunoblotted for TDP-43 or HuR. **B–C**: Cells were treated for 2 hr with paraquat in the presence or absence of 1 µM Cu^II^(atsm), 1 µM CuCl_2_ or 10 µM PD98059 (ERK inhibitor). Cells were immunoblotted for phospho-ERK (p-ERK), total ERK, phospho-JNK (p-JNK) and total JNK. **D–E**: Cells were treated overnight with paraquat in the presence or absence of 1 µM Cu^II^(atsm), 1 µM CuCl_2_ or 10 µM PD98059 (ERK inhibitor). Cells were immunoblotted for phospho-ERK (p-ERK), total ERK, phospho-JNK (p-JNK) and total JNK. **F**: Effect of CuCl_2_ on ERK and JNK phosphorylation. Cells were treated overnight with 1 mM paraquat or paraquat plus 100 µM CuCl_2_ and cells were examined for expression ERK and JNK phosphorylation. **G:** HeLa cells were treated for 1 hr with 500 µM sodium arsenite in presence or absence of 1 µM Cu^II^(atsm). Cells were immunoblotted for phospho-ERK (p-ERK), total ERK, phospho-JNK (p-JNK) and total JNK. *p<0.05 compared to untreated control. **p<0.05 compared to treatment alone.

### Immunofluorescence Analysis

SH-SY5Y cells were grown on 15 mm diameter coverslips and treated as indicated. Cells were fixed with 4% w/v paraformaldehyde in PBS for 30 min and permeabilized with 90% chilled methanol for 5 min. After blocking for 1 hr with 10% normal goat serum, cells were incubated with primary antibody (for total TDP-43 (1∶1,500), ubiquitin (1∶150) or HuR (1∶50), for 2 hr at room temperature or overnight at 4°C. This was followed by labeling with secondary AlexaFluor or FITC goat anti-mouse or anti-rabbit antisera at 1∶500 for 2 hr at room temperature or overnight at 4°C. After washing, the coverslips were incubated with DAPI at 0.5 ng/ml for 5 min and analyzed using a Leica inverted microscope with Zeiss Axiocam digital camera. Images shown are representative of multiple fields and triplicate coverslips per experiment. TDP-43 and HuR-positive stress granules (SGs) were counted in cultures where indicated. A minimum of 500 cells was counted across multiple fields of view (and multiple coverslips) for each treatment. The number of TDP-43 and HuR-positive SGs were counted in these cells. The total number of cells was divided by the total number of SGs to provide a measure of mean SGs per cell. SGs were not observed in untreated cells.

### Preparation of TDP-43 Plasmids

Plasmid DNA corresponding to GFP-tagged full-length wild-type (WT) TDP-43 (pEGFP-TDP WT) and the C-terminal fragment of TDP-43, (pEGFP-TDP 219–414) or empty expression vector pEGFP-C1 were prepared as described by Nonaka et. al. [14], [22]. Briefly, plasmid DNA was used to transform MAX Efficiency® DH5α™ Competent Cells (Invitrogen, Mount Waverley, Victoria, Australia) as described by the manufacturer. Transformants were grown and colonies were picked based on kanamycin-resistance and grown in liquid culture for subsequent plasmid purification. DNA was purified using the Wizard® *Plus* Midiprep DNA Purification System (Promega Corporation) as per manufacturer’s instructions. DNA was quantified and TDP-43 inserts were identified positively by digestion with *BamHI* and *XhoI.*

### Transfection and Expression of Plasmids

SH-SY5Y cells were seeded at 2×10^51^ cells per well in 24 well–plates on coverslips. Cells were transfected 24 hr after seeding with the pEGFP-C1 empty vector, pEGFP-TDP WT and pEGFP-TDP 219–414 using Dreamfect (Qiagen) according to manufacturer’s instructions. After 48 hr incubation, cells were fixed with 4% w/v paraformaldehyde in PBS for 30 min and permeabilized with 90% chilled methanol for 5 min. After washing, the coverslips were incubated with DAPI at 0.5 ng/ml for 5 min and analyzed using a Leica inverted microscope with Zeiss Axiocam digital camera. Expression of TDP-43 was determined by the EGFP-tagged construct. Efficiency of transfection with pEGFP-C1 vector was approximately 20–25% [22].

**Figure 5:**
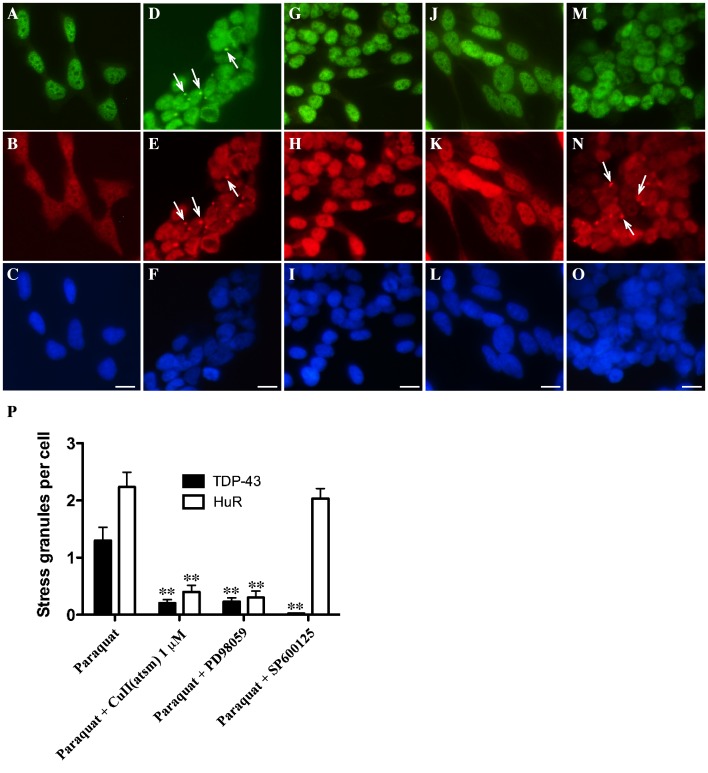
Effect of Cu^II^(atsm) and kinase inhibitors on TDP-43 and HuR-positive SG formation. Cells were treated overnight with 1 mM paraquat in the presence or absence of 1 µM Cu^II^(atsm), 10 µM PD98059 or 20 µM SP600125 and examined for TDP-43 and HuR by immunofluorescence. **A–C**: untreated, **D–F**: paraquat, **G–I**: paraquat and Cu^II^(atsm), **J–L**: paraquat and PD98059, **M–O**: paraquat and SP600125. Green  =  TDP-43, red  =  HuR, blue  =  DAPI. Arrows indicate TDP-43 and HuR SGs. Bar  = 10 µm. **P**: SGs (TDP-43 or HuR-positive) per cell after overnight treatment with 1 mM paraquat and co-treatment with Cu^II^(atsm) (1 µM), PD98095 or SP600125. **p<0.01 compared to paraquat treatment.

### Statistical Analysis

All data described in graphical representations are mean ± standard error of the mean (SEM) from a minimum of three experiments. Densitometric analysis of immunoblots was performed using NIH ImageJ 1.43 software on two-three separate blots and adjusted for loading control (GAPDH or actin). Results were analysed using a two-tailed Student’s *t-*test and ANOVA where appropriate.

## Results

### Cu^II^(btsc)s Inhibit TDP-43-positive SGs Induced by a Mitochondrial Inhibitor

To induce TDP-43-positive SGs, SH-SY5Y cells were exposed overnight to 1 mM paraquat (mitochondrial inhibitor). As reported previously, this exposure to paraquat induces only mild loss of cell viability (∼15% as determined by MTT assay) and no cell death (as determined by LDH assay) [22]. We confirmed that paraquat treatment induced mitochondrial inhibition through analysis of ATP levels in treated cells (Figure S1). Consistent with our previous study, the paraquat treatment induced frequent TDP-43 and HuR-positive SGs in the cytosol of treated cells together with loss of nuclear TDP-43 in some cells (Figure 1E–H). Cells were co-treated with 1 µM Cu^II^(atsm) (Figure 1I–L) or 50 nM Cu^II^(gtsm) (Figure 1M–P). These concentrations were selected as the maximum doses that did not induce a significant increase in cell death (LDH release) after overnight exposure. Cu^II^(gtsm) has a higher potential to induce toxicity than Cu^II^(atsm) due to the fact that Cu^I^ is readily released in the intracellular reducing environment from Cu^II^(gtsm) [27]. Cu^II^(atsm) is more resistant to reduction of Cu^II^ to Cu^I^ under normoxic conditions and therefore is thought to release less Cu^I^ [23]. Hence Cu^II^(gtsm) was used at a concentration 20-fold less than Cu^II^(atsm) due to its higher potential to release bioavailable Cu.

**Figure 6:**
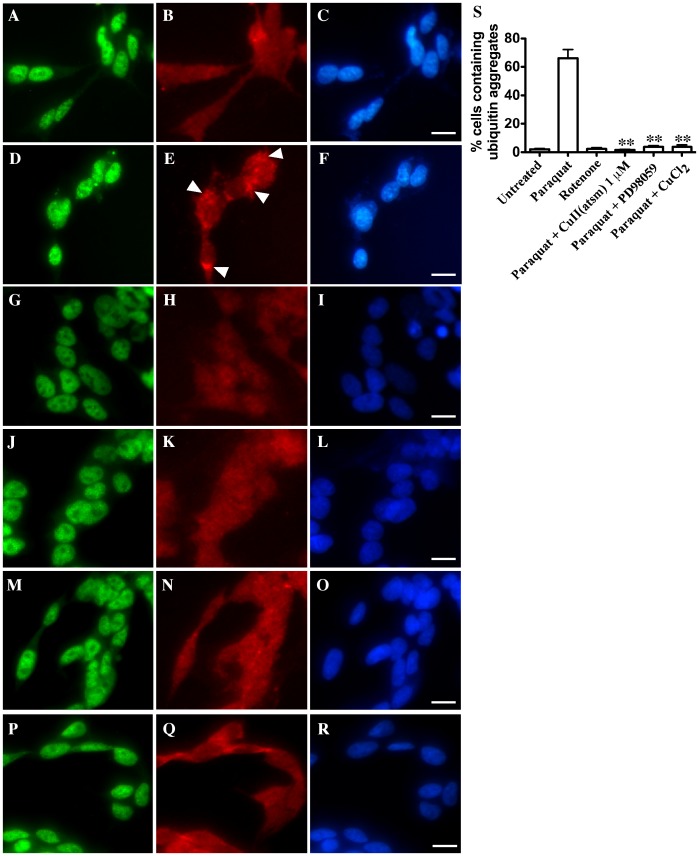
Effect of Cu^II^(atsm) on paraquat-mediated changes to ubiquitin. Cells were treated with 1 mM paraquat overnight in the presence or absence of 1 µM Cu^II^(atsm), 10 µM PD98059 or 100 µM CuCl_2_. Cells were also exposed overnight to 75 µM rotenone. Cells were examined for TDP-43 and ubiquitin by immunofluorescence. **A–C**: untreated, **D–F**: paraquat, **G–I**: rotenone, **J–L**: paraquat and Cu^II^(atsm), **M–O**: paraquat and PD98059 (ERK inhibitor), **P–R**: paraquat and CuCl_2_. Green  =  TDP-43, red  =  ubiquitin, blue  =  DAPI. Arrowheads indicate ubiquitin aggregation. Bar  = 10 µm. **S**: Percentage of cells containing ubiquitin aggregates after overnight treatment with 1 mM paraquat, 75 µM rotenone, paraquat plus 1 µM Cu^II^(atsm), paraquat plus PD98059 or paraquat plus 100 µM CuCl_2_. **p<0.01 compared to paraquat treatment.

Co-treatment with either Cu^II^(atsm) or Cu^II^(gtsm) substantially inhibited formation of TDP-43 and HuR-positive SGs induced by paraquat (Figure 1I–L and M–P). To determine the specificity of this effect, we compared Cu^II^(atsm) and Cu^II^(gtsm) with CuCl_2_ at 1 µM and 100 µM and FeCl_2_ at 100 µM. ZnCl_2_ was not examined as we have reported previously that ZnCl_2_ induces nuclear aggregates of TDP-43 [30]. Co-treatment of SH-SY5Y cells with paraquat and 1 µM CuCl_2_ had no effect on TDP-43 or HuR SGs (Figure 2G–H), however, 100 µM CuCl_2_ induced an effect indistinguishable to 1 µM Cu^II^(atsm) and 50 nM Cu^II^(gtsm) (Figure 2I–J and Figure 2M). These findings are consistent with the fact that Cu^II^(btsc)s are highly efficient at delivering intracellular Cu when compared to CuCl_2_ and therefore a higher level of CuCl_2_ is required to obtain the same increase in cellular bioavailable Cu [24], [27]. In contrast to Cu, changes to paraquat-mediated TDP-43 or HuR SGs were not detected in cultures co-treated with FeCl_2_ at 100 µM (Figure 2K–L). Similarly, treatment of cells with the metal-free gtsmH_2_ ligand had no effect on SG formation (data not shown). These findings demonstrated that co-treatment of SH-SY5Y neurons with low concentrations of Cu^II^(btsc)s inhibited paraquat-mediated SG formation, including TDP-43-positive SGs and this was likely to be related to the ability of the Cu^II^(btsc)s to efficiently deliver bioavailable Cu into the cell. Subsequent studies were performed using Cu^II^(atsm) due to the significant potential of this compound as a therapeutic agent for ALS based on efficacy in our preliminary *in vivo* studies [25], [26].

Next we examined if paraquat-mediated TDP-43-positive SGs could be inhibited in HeLa cells. Co-exposure of HeLa cells with 1 µM Cu^II^(atsm) and 1 mM paraquat overnight blocked formation of SGs as observed in SH-SY5Y cells (data not shown). To determine if Cu^II^(atsm) could inhibit against TDP-43-positive SGs generated by another common inhibitor of mitochondrial function, we treated HeLa cells with 500 µM sodium arsenite for 1 hr. This is the most common method used for SG induction and we have shown previously that sodium arsenite induces robust TDP-43 and HuR SGs in HeLa cells. However, co-treatment with 1 µM Cu^II^(atsm) for 1 hr, 4 hr, or overnight prior to the 1 hr sodium arsenite treatment had no effect on the formation of TDP-43 and HuR-positive SGs (Figure 3). The reason for this was not known but may have been related to the short time frame of SG induction with sodium arsenite (1 hr) compared to the longer-term induction with paraquat (overnight).

**Figure 7:**
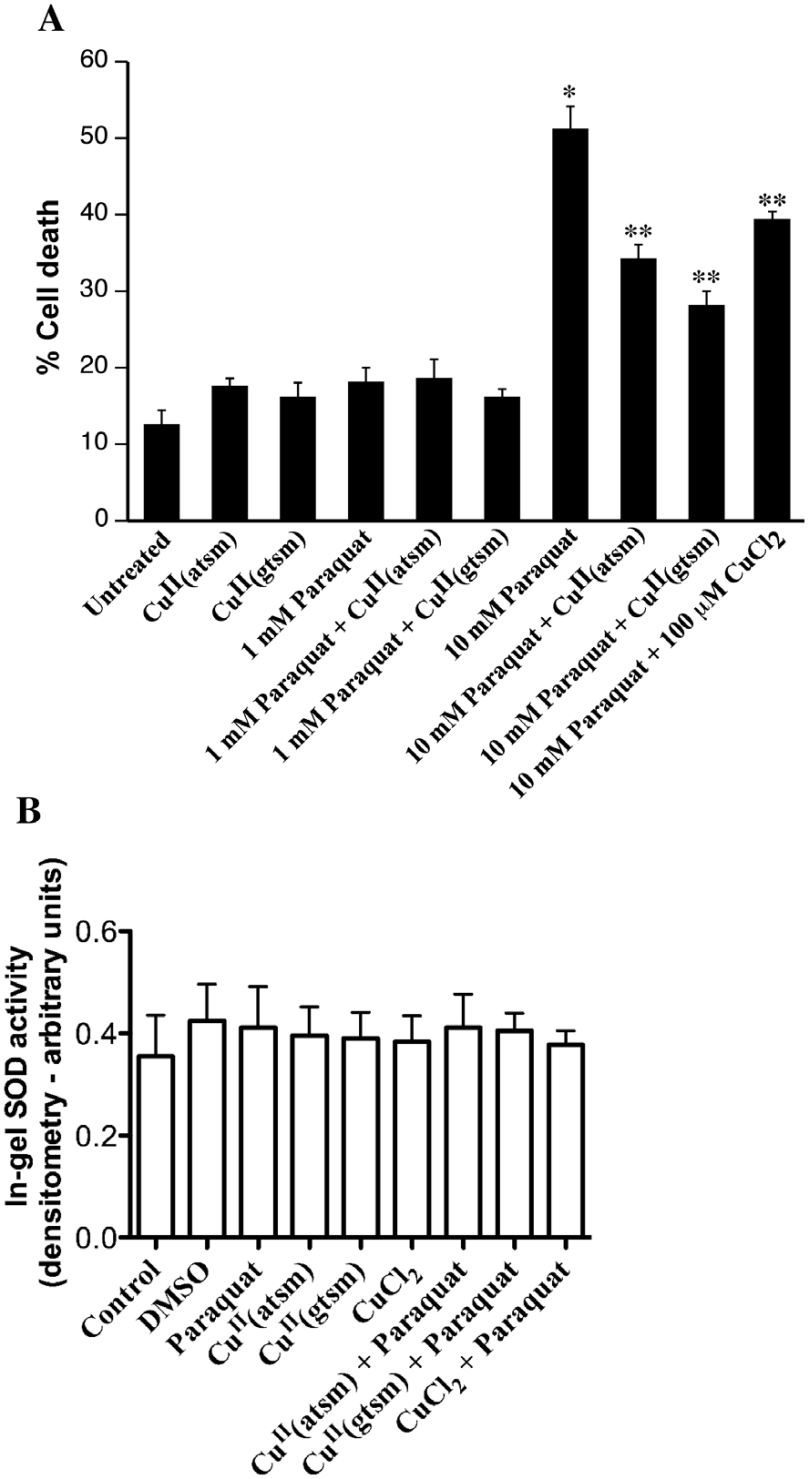
Effect of Cu^II^(btsc)s on paraquat toxicity. **A**: Cells were treated overnight with 1 or 10 mM paraquat in the presence or absence of 1 µM Cu^II^(atsm), 50 nM Cu^II^(gtsm) or 100 µM CuCl_2_ and cell death was measured with the LDH assay. *p<0.01 compared to untreated cells. **p<0.01 compared to 10 mM paraquat alone. **B**: In-gel SOD activity assay. Cells were treated with 1 mM paraquat overnight alone or in the presence of 1 µM Cu^II^(atsm), 50 nM Cu^II^(gtsm) or 1 µM CuCl_2_. Cu^II^(atsm), Cu^II^(gtsm) and CuCl_2_ were also added at the same concentration to cultures without paraquat. Cell lysates were analyzed for SOD activity using a SOD zymography gel assay. The band representing SOD activity was analyzed by densitometry and revealed no significant change compared to untreated control.

### Cu^II^(atsm) did not Inhibit Expression of TDP-43 or HuR

To examine how Cu^II^(atsm) inhibited TDP-43 and HuR-positive SGs, we first determined if there was any effect on TDP-43 or HuR expression. Cells treated with 1 µM Cu^II^(atsm) alone overnight revealed no substantial alterations in full length TDP-43 or HuR expression (data not shown). Interestingly whilst paraquat induced a slight increase in 35 kDa TDP-43 (Figure 4A), co-exposure with 1 µM Cu^II^(atsm) further increased this expression (Figure 4A). Using N-terminal and C-terminal antibodies, we have confirmed that the 35 kDa TDP-43 is a C-terminal fragment as expected (data not shown). Cu^II^(atsm) did not affect 35 kDa CTF-TDP-43 levels in the absence of paraquat treatment (data not shown). The effect on 35 kDa CTF-TDP-43 was not observed with 1 µM CuCl_2_ (Figure 4A), although 100 µM CuCl_2_ did slightly increase the expression of the 35 kDa band (Figure S2A). We reported previously that inhibition of JNK with SP600125 also leads to elevated 35 kDa CTF-TDP-43 [22]. As those studies were performed in conjunction with the present analysis, we have included the combined results in Figure 4A to demonstrate that both JNK inhibition and treatment with Cu^II^(atsm) lead to elevated 35 kDa CTF-TDP-43 levels. HuR expression was not substantially altered in any condition tested (Figure 4A). These results showed that the inhibition of TDP-43/HuR-positive SGs was not due to loss of expression of either protein by Cu^II^(atsm).

**Figure 8:**
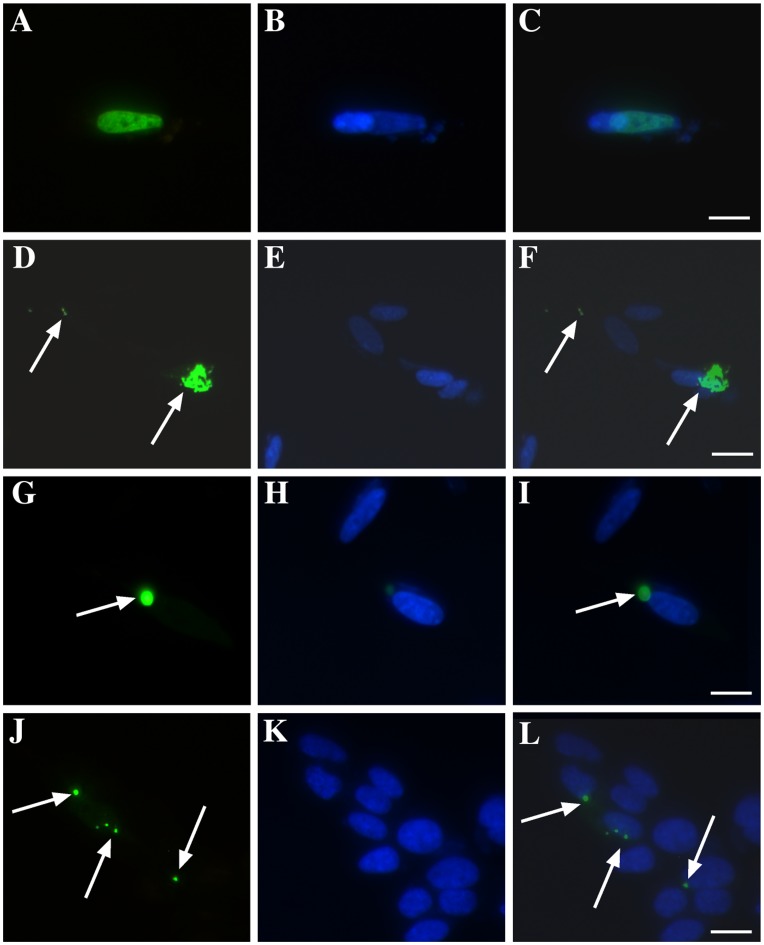
Effect of Cu^II^(atsm) on aggregation of CTF-TDP-43 219–414. Cells were transfected with WT TDP-43-GFP (full length) or CTF-TDP-43 219–414-GFP and formation of TDP-43 aggregates was examined after 48 hr. **A–C**: Aggregates of TDP-43 were not observed in cells transfected with WT-TDP-43. **D–F**: Upon transfection with CTF-TDP-43 219–414, cells revealed widespread formation of cytosolic aggregates after 48 hr. **G–I**: Addition of Cu^II^(atsm) (1 µM) to cells at 24 hr resulted in a reduction in frequency of cells expressing CTF-TDP-43 219–414 aggregates at 48 hr. **J–L**: Addition of CuCl_2_ (1 µM) to cells at 24 hr resulted in no significant change to the frequency of cells expressing CTF-TDP-43 219–414 aggregates at 48 hr. C, F, I and L represent merged images of TDP and DAPI. Arrows indicate TDP-43 aggregates. Green  =  TDP-43, blue  =  DAPI. Bar  = 10 µm.

### Cu^II^(atsm) Inhibits ERK-mediated SG Formation

We further investigated the effect of Cu^II^(atsm) on kinases. As reported previously [22], [36], paraquat induced significant ERK and JNK phosphorylation after 30 min with maximal expression at 2–4 hr and this was sustained overnight. Moreover, we also reported that ERK inhibition blocked TDP-43 and HuR-positive SG formation while JNK inhibition blocked only TDP-43 accumulation in SGs [22], [36]. Therefore, we examined if Cu^II^(atsm) inhibited ERK activation (Figure 4B). Co-treatment of cells with Cu^II^(atsm) did not inhibit levels of phosphorylated ERK when examined after 2 hr of incubation (Figure 4B). An analogous result was observed for JNK activation, with Cu^II^(atsm) having no effect on JNK phosphorylation after 2 hr incubation with paraquat (Figure 4C). In contrast, after overnight incubation with 1 µM Cu^II^(atsm), both ERK and JNK phosphorylation were significantly inhibited compared to paraquat alone (Figure 4D and E). Cu^II^(atsm) alone had no effect on basal ERK or JNK phosphorylation over the same time period (Figure S2B). CuCl_2_ at 1 µM had no effect on paraquat-mediated kinase phosphorylation (Figure 4D), although at 100 µM, inhibition of paraquat-mediated ERK and JNK phosphorylation was observed (Figure 4F).

**Table 1:** Effect of Cu^II^(atsm) or CuCl_2_ on frequency of cells expressing CTF-TDP-43 219–414 aggregates.

TDP-43 construct	Cells expressing aggregates^1^
CTF-TDP-43 219–414	443±38 (100%)
CTF-TDP-43 219–414+ Cu^II^(atsm) (added at time of transfection)	296±42 (66.8%) (P<0.01)
CTF TDP-43 219–414+ Cu^II^(atsm) (added at 24 hr post-transfection)	226±32 (51.0%) (P<0.01)
CTF TDP-43 219–414+1 µM CuCl_2_ (added at 24 hr post-transfection)	408±27 (92.1%)
CTF TDP-43 219–414+100 µM CuCl_2_ (added at 24 hr post-transfection)	263±36 (59.3%)
WT TDP-43	None observed

1 Per 1,000 GFP-positive transfected cells.

We also examined the effect of Cu^II^(atsm) on sodium arsenite-treated HeLa cells. Exposure of HeLa cells to 500 µM sodium arsenite for 1 hr induced significant activation of JNK and ERK (Figure 4G). Co-exposure to Cu^II^(atsm) did not substantially affect activation of either kinase (Figure 4G), which was consistent with the lack of inhibition of SG formation by Cu^II^(atsm) in sodium arsenite-treated cells (Figure 3).

As Cu^II^(atsm) was found to inhibit ERK phosphorylation in SH-SY5Y cells treated with paraquat, we compared the effect of Cu^II^(atsm) and an ERK inhibitor on SG formation. We found that both Cu^II^(atsm) and PD98059 (ERK inhibitor) induced the same inhibition of TDP-43 and HuR-positive SGs when co-treated with paraquat (compare Figure 5G–I (Cu^II^(atsm) with Figure 5J–L (PD98059)) and also Figure 5P. This study was performed in parallel with our previous investigation showing that JNK inhibition blocked formation of TDP-43-positive, but not HuR-positive, SGs [22], [36]. Here, we present comparative data from the study to show that while Cu^II^(atsm) has an analogous effect to inhibition of ERK, the results contrast with the more specific action of JNK inhibition on TDP-43 alone (Figure 5M–O). These findings suggested that Cu^II^(atsm) could inhibit TDP-43 and HuR-positive SG formation through inhibition of prolonged ERK activation. This is consistent with our observations that paraquat does not induce TDP-43/HuR-positive SGs until after 8 hr of exposure [22]. This also explains why Cu^II^(atsm) did not block SGs induced by 1 hr treatment with sodium arsenite, as prolonged exposure to Cu^II^(atsm) e.g. overnight in the presence of the mitochondrial inhibitor, is needed to obtain a protective response from the copper complex.

### Cu^II^(atsm) Inhibits Ubiquitin Accumulation Induced by Paraquat

Due to the observation that Cu^II^(atsm) only inhibited ERK (and JNK) activation after a prolonged exposure with paraquat, we further investigated the potential neuroprotective mechanism of Cu^II^(atsm) that might account for this. We examined the effect of Cu^II^(atsm) on paraquat-induced changes to ubiquitin. Previous reports have demonstrated that paraquat treatment leads to altered proteasomal function and can result in accumulation of ubiquitinated proteins [37], [38]. This stress may lead to SG formation [39], [40]. In our cultures, we also found that treatment with paraquat induced robust abnormal accumulation of ubiquitin in the cytosol of treated cells (Figure 6D–F) and Figure 6S. Higher order ubiquitinated aggregates were not observed by immunoblot analysis (data not shown). This indicated that the changes to cellular ubiquitin accumulation were probably not associated directly with protein aggregation but more likely a redistribution of ubiquitinated proteins in the cytosol. Although the majority of the ubiquitin accumulation was not directly co-localized with TDP-43 SGs (Figure 6D compared to Figure 6E), ubiquitin accumulation was not induced by alternative mitochondrial inhibitors that failed to cause SG formation such as rotenone (Figure 6G–I) or MPP+ (not shown). As only paraquat induced both ubiquitin accumulation and SGs, these data supported a potential association between altered SG formation and abnormal ubiquitinated protein distribution. Co-treatment with 1 µM Cu^II^(atsm) resulted in complete abrogation of the ubiquitin accumulation in paraquat-treated cultures (Figure 6J–L and Figure 6S). Moreover, co-treatment of cultures with the ERK inhibitor, PD98059 prevented the accumulation of ubiquitin induced by paraquat (Figure 6M–O and Figure 6S). As expected, 100 µM CuCl_2_ also prevented accumulation of ubiquitin (Figure 6P–R and Figure 6S). This was consistent with previous studies, which report that adequate Cu homeostasis is associated with normal ubiquitin metabolism [41] and suggest a potential link between Cu and ERK activity in this action. Moreover, the results indicated that Cu^II^(atsm) may have a broad neuroprotective effect leading to decreased cell stress and therefore lack of SG formation.

### Cu^II^(atsm) Protects Against Paraquat Neurotoxicity

To confirm this protective action, we measured the ability of Cu^II^(atsm) to protect against paraquat-induced cell death. As 1 mM paraquat did not induce substantial cell death, we exposed cultures to 10 mM paraquat overnight (Figure 7A). This treatment induced a significant increase in cell death from 12% to 52% (Figure 7A). Co-treatment with 1 µM Cu^II^(atsm) significantly reduced this neurotoxic action to 35% (Figure 7). In addition, Cu^II^(gtsm) (50 nM) also significantly decreased paraquat-mediated toxicity (Figure 7A) (28%). As expected, 100 µM CuCl_2_ had a significant inhibitory effect on paraquat neurotoxicity. As the use of a Cu^II^-complex could potentially inhibit superoxide-mediated cell stress by a superoxide dismutase (SOD)-like activity, we examined the SOD activity using an in-gel assay. Exposure of SH-SY5Y cells to 1 µM Cu^II^(atsm), 50 nM Cu^II^(gtsm) or 1 µM CuCl_2_ in the presence or absence of 1 mM paraquat resulted in no significant increase in SOD-like activity in treated cells as compared to untreated controls (Figure 7B). These findings are consistent with a neuroprotective action of Cu^II^(btsc)s mediated through inhibition of protein accumulation and kinase activation rather than through enhanced SOD or SOD-like activity.

### Cu^II^(atsm) Inhibits Aggregation of CTF-TDP-43

Finally, although we had shown that Cu^II^(atsm) could inhibit endogenous TDP-43-containing SGs induced by mitochondrial inhibition, we were interested to know if this action could be extended to prevention of pathological aggregation of TDP-43 associated with ALS. To investigate this, we transfected SH-SY5Y cells with wild-type (WT) full length TDP-43-GFP and a C-terminal fragment known to aggregate in cultured neurons (CTF-TDP-43 219–414-GFP) [22]. As previously reported, transfection of cells with WT full length TDP-43 did not result in aggregation of TDP-43 after 48 hr (Figure 8A–C). In contrast, transfection with CTF-TDP-43 219–414 for 48 hr resulted in robust formation of cytosolic TDP-43 aggregates (Figure 8D-F). Cells were co-treated with 1 µM Cu^II^(atsm) either at the time of CTF-TDP-43 219–414 transfection or 24 hr later. At 48 hr after transfection, cells were assessed for the level of CTF-TDP-43 219–414 aggregation. Cu^II^(atsm) significantly reduced the number of TDP-43 aggregates in transfected cells when added at time of transfection (0 hr) or at 24 hr post-transfection (Figure 8 and Table 1). CuCl_2_ at 1 µM had no effect on the numbers of cells with aggregates, although 100 µM CuCl_2_ did induce inhibition of aggregate formation (Table 1). Interestingly, Cu^II^(atsm) had a greater inhibitory effect when added after 24 hr, reducing the number of cells with aggregates by ∼50% (Table 1). This may be due to loss of Cu^II^(atsm) from the cell before accumulation of CTF-TDP-43 219–414 when added at time of transfection. Most aggregates form in the second 24 hr after transfection. As we have shown previously that JNK inhibition, but not ERK inhibition, significantly inhibits accumulation of CTF-TDP-43 219–414 [22], it is perhaps more likely that Cu^II^(atsm) inhibition of CTF-TDP-43 aggregation here is mediated through its inhibitory effect on JNK (associated with cell stress induced by CTF-TDP-43 aggregation). However, these studies show that Cu^II^(atsm) can significantly reduce cellular accumulation of both endogenous and transfected TDP-43.

## Discussion

In this study, we investigated the ability of Cu^II^(btsc) Cu-complexes to inhibit stress-mediated TDP-43-positive SGs in neuronal cell culture. We found that Cu^II^(atsm) and Cu^II^(gtsm) inhibited paraquat-mediated sustained ERK phosphorylation resulting in abrogation of SG formation and improved survival of cells treated with paraquat. In addition, both Cu^II^(atsm) and the ERK inhibitor, PD98059, inhibited accumulation of ubiquitin in paraquat-treated cells indicating that this may be a protective action of Cu^II^(atsm) mediated through inhibition of prolonged ERK activation. Cu^II^(atsm) also reduced aggregation of CTF-TDP-43 in transfected cells.

Cu^II^(btsc)s have been investigated extensively as hypoxia imaging agents and as potential anti-cancer compounds for delivery of radionuclides to tumors [23]. The value of these compounds is based on their ability to modify intracellular Cu release based on subtle modification of the ligand backbone structure. Cu^II^(atsm) has a relatively negative Cu^II^/^I^ reduction potential (*E^0’^* = −0.59 V vs SCE) and this limits reduction of the metal ion to Cu^I^ and the subsequent release of bioavailable Cu^I^ into cells under normal cell conditions but Cu^I^ release is enhanced under hypoxia, cellular energy impairment and probably within select sub-cellular compartments within the cell [23], [27], [35]. In contrast, in the case of Cu^II^(gtsm), it is easier to reduce the Cu^II^ to Cu^I^ (*E^0’^* = −0.43 V vs SCE) so the complex is able to release considerable Cu^I^ under basal cellular reducing conditions. In previous studies, we have harnessed these properties to develop Cu^II^(btsc)s as potential therapeutic agents to deliver small levels of bioavailable Cu to the brain. This is achieved through the ability of these compounds to cross the blood brain barrier [42]. We have shown in previous studies that Cu^II^(btsc)s can modulate cell signaling pathways including phosphorylation and activity of phosphoinositol-3-kinase (PI3K), ERK, glycogen synthase kinase 3 (GSK3) and JNK [24], [27], [29]. The ability to control these kinases through Cu release may be central to the neurotherapeutic effects we have observed using Cu^II^(btsc)s [24]–[26]. In a previous study, we have found that Cu^II^(atsm) significantly delayed disease onset and extended life span in a low transgene copy SOD1G93A murine model of ALS. We observed that Cu^II^(atsm) delayed disease onset, extended survival and inhibited oxidative damage and glial activation in treated mice [26]. While we have reported evidence for a potential anti-peroxynitrite action of Cu^II^(atsm), the therapeutic action of this complex in the SOD1G93A mice remains unknown [26]. Interestingly, Cu^II^(atsm) did inhibit accumulation of phosphorylated full length and C-terminal TDP-43 in the treated SOD1G93A mice. Our data here and previously [22] suggest that this effect could be related to inhibition of kinase activity associated with pathological changes to TDP-43. However, we have not observed significant phosphorylation of endogenous TDP-43 in our cell culture models of impaired TDP-43 processing. In addition, Cu^II^(atsm) has also protected against neurodegeneration and improved motor performance in models of Parkinson’s disease [25], suggesting that the metal-complex may have broad neuroprotective action. In the present study, we examined the effect of this class of compound on SG formation, and in particular, the effect on TDP-43 association with SGs.

We previously reported that treatment of neuronal and non-neuronal cell cultures with paraquat overnight induced a robust induction of TDP-43-positive SGs and that this was mediated through ERK and JNK activation [22]. Whether TDP-43-positive SG formation is an important early indicator of abnormal TDP-43 processing and accumulation relevant to ALS is not yet known. However, studies have shown that pathogenic TDP-43 C-terminal fragments co-localize with SG markers in cells [15], [16], [18], [19], [43] and TDP-43 SGs have been observed in ALS spinal tissue [16]. Moreover, FUS, another protein associated with ALS and a member of the hnRNP family (as is TDP-43) also co-localizes to SGs in cells and ALS tissue [20], [21]. These studies suggest that SG formation by TDP-43 may precipitate further aggregation and abnormal metabolism of TDP-43. Therefore, preventing TDP-43 SG formation may be a logical therapeutic target. As inhibition of mitochondrial function is an important feature of ALS and other neurodegenerative diseases, we were interested in whether Cu^II^(btsc)s could inhibit formation of TDP-43-positive SGs induced by the mitochondrial inhibitor, paraquat.

The ability of Cu^II^(atsm) to inhibit TDP-43-and HuR-positive SG formation during paraquat-mediated stress was likely due to its ability to inhibit ERK phosphorylation. Although Cu^II^(atsm) also inhibited prolonged JNK phosphorylation, we have shown that inhibition of JNK in this model stops TDP-43 association with SGs but not HuR SG formation. In this study, Cu^II^(atsm) and the ERK inhibitor, PD98059, both prevented formation of HuR-and TDP-43-positive SGs making the inhibition of ERK potentially more important to these protective effects than JNK inhibition in this cell model. However, the JNK inhibitory action of Cu^II^(atsm) was perhaps more likely to be involved in the reduced aggregation of CTF-TDP-43 219–414 in transfected cells. This is because we reported previously that JNK, but not ERK inhibition, reduced accumulation of CTF-TDP-43 219–414 and 162–414 in the same cell type [22]. Alternatively, Cu^II^(atsm) could mediate inhibition of CTF-219–414 TDP-43 aggregation through an unrelated mechanism.

Interestingly, Cu^II^(atsm) did not inhibit ERK or JNK phosphorylation after 2–4 hr exposure to the Cu-complex but did inhibit both kinases after an overnight treatment. These findings suggested that Cu^II^(atsm) had a delayed effect and prevented prolonged activation. This may be due to inhibition of complex upstream pathways and could be associated with the prevention of ubiquitin accumulation observed in cultures treated with paraquat. The fact that both ERK inhibition and Cu^II^(atsm) blocked the ubiquitin aggregation suggested that ERK inhibition is upstream of changes to ubiquitin in paraquat-treated cells. Interestingly, other mitochondrial inhibitors did not induce effects on ubiquitin or induce SGs supporting a close connection between ubiquitin changes and SG formation, possibly controlled by ERK. It has been reported elsewhere that ERK (and JNK) have important roles in the toxic effects of paraquat [38], [44]–[46].

How Cu^II^(atsm) is inhibiting ERK is not known but is likely to involve release of Cu as we observed the same effect with Cu^II^(gtsm), which is known to release intracellular Cu. We have also found Cu^II^(gtsm) to potently inhibit ERK at low concentrations in other cell lines including PC12 neurons and primary cortical neurons (data not shown). Interestingly, we have shown that under certain conditions, Cu^II^(atsm) can also activate ERK rather than inhibit its activity [35]. While the current results showing Cu^II^(atsm)-mediated inhibition of ERK may initially appear contradictory, it is likely that the different outcomes of Cu^II^(atsm) treatment are related to basal ERK activity in each model system. Our unpublished studies have shown that if ERK phosphorylation is low, Cu^II^(atsm) (under conditions of energy inhibition) can increase phosphorylation, whereas if ERK phosphorylation is already high (as in paraquat-treated cultures in this study) Cu^II^(atsm) can inhibit ERK. The reason for this dynamic effect is not known but may be related to changes in subcellular Cu pools.

It has been reported elsewhere that Cu can enhance function of ubiquitin-associated activities, although excess Cu can also induce abnormal ubiquitination [47]. Our results suggest that delivery of low levels of Cu to cells under stress may enhance protein turnover and relieve accumulation of proteins and prevent cell stress, but excess Cu delivery would be toxic. The protective effects of Cu^II^(atsm) and Cu^II^(gtsm) in this system were supported by our finding that both compounds inhibited cell death induced by paraquat.

While Cu^II^(atsm) inhibited paraquat-induced TDP-43 and HuR SGs, there was no effect on SGs formed by sodium arsenite treatment. The reason for this may be the time frame required for the Cu-complex to act. We tested Cu^II^(atsm) against sodium arsenite by using pre-treatment regimes of overnight or 4 hr but these had no effect, indicating that the Cu^II^(atsm) should be present at the same time as the stress and that the effect is then mediated through a relatively slow action. Whether this is related to Cu release from the complex under the paraquat-induced stress or the need for Cu to induce protein synthesis is unknown. However, as ALS is a slowly progressing disease, these complexes may have therapeutic potential. We have shown that Cu^II^(atsm) inhibits disease progression in a SOD1 model of ALS [26]. Although effects in SGs have not been investigated, cell studies have shown that mutant SOD1 may be associated with SGs. In addition, we found that Cu^II^(atsm) inhibited formation of phosphorylated cleavage fragments of TDP-43 in the treated SOD1 mice [26]. In our paraquat-treated cells, we have not observed changes to phosphorylation of TDP-43, suggesting that this may be a later effect associated with advanced disease as suggested previously [22]. It will be of interest to determine if Cu^II^(atsm) protects mice from disease induced by overexpression or mutation of TDP-43 [48], and whether this is related to changes to TDP-43 SG formation early in disease.

In summary, we have found that co-treatment of cells with Cu^II^(bstc) complexes can inhibit formation of TDP-43-positive SGs and prevent paraquat-mediated cell death. This neuroprotection appeared to be associated with inhibition of ERK phosphorylation and may be mediated through modulation of cellular ubiquitination by Cu. Further studies are warranted to determine if Cu^II^(atsm) or related Cu^II^(btsc)s are potential therapeutics for ALS and other neurodegenerative diseases through kinase modulation and maintenance of ubiquitin homeostasis.

## Supporting Information

Figure S1 **ATP levels in paraquat-treated cells. SH-SY5Y cells were treated overnight with 1 mM paraquat and total cellular ATP levels were determined in cell lysates.** *p<0.05 compared to control cells.
(TIF)

Figure S2 **Effect of CuCl_2_ on TDP-43 expression.** **A**: Cells were treated with 1 mM paraquat overnight in the presence or absence of 1 µM Cu^II^(atsm) or 100 µM CuCl_2_. Cells were examined for expression of full length TDP-43 and 35 kDa CTF-TDP-43 by western blot. **B**: Effect of Cu^II^(atsm) on ERK and JNK phosphorylation. Cells were treated overnight with 1 µM Cu^II^(atsm) and examined for expression of phosphorylated ERK and JNK.
(TIF)
