# Supplementary figures and images for: Inhibition of TDP-43 Accumulation by Bis(thiosemicarbazonato)-Copper Complexes
Source: PLoS One. 2012 Aug 3;7(8):e42277. doi: 10.1371/journal.pone.0042277 (PMC3411774; doi:10.1371/journal.pone.0042277)

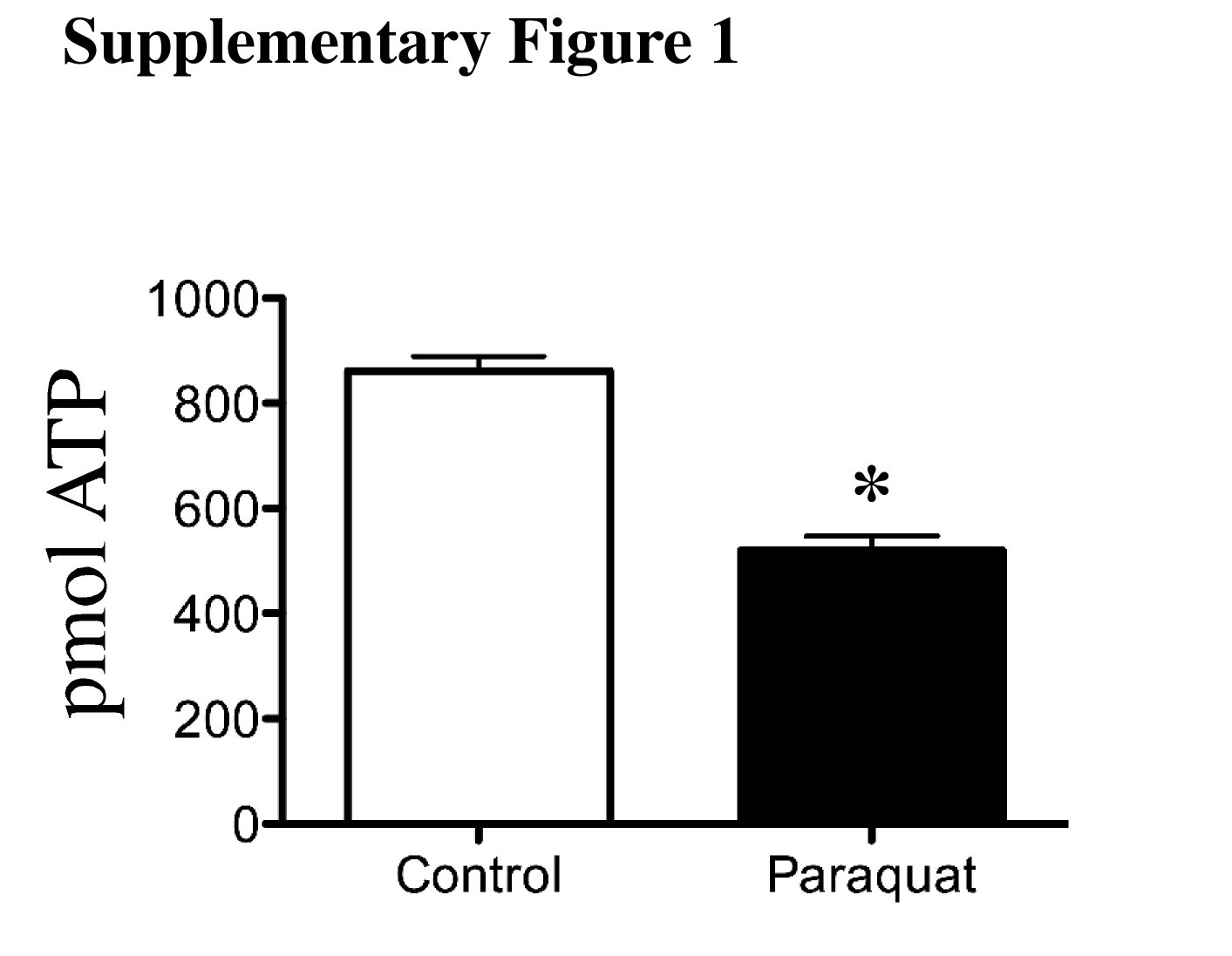

Supplement: Figure S1 — ATP levels in paraquat-treated cells. SH-SY5Y cells were treated overnight with 1 mM paraquat and total cellular ATP levels were determined in cell lysates. *p<0.05 compared to control cells. (TIF) [file pone.0042277.s001.tif]

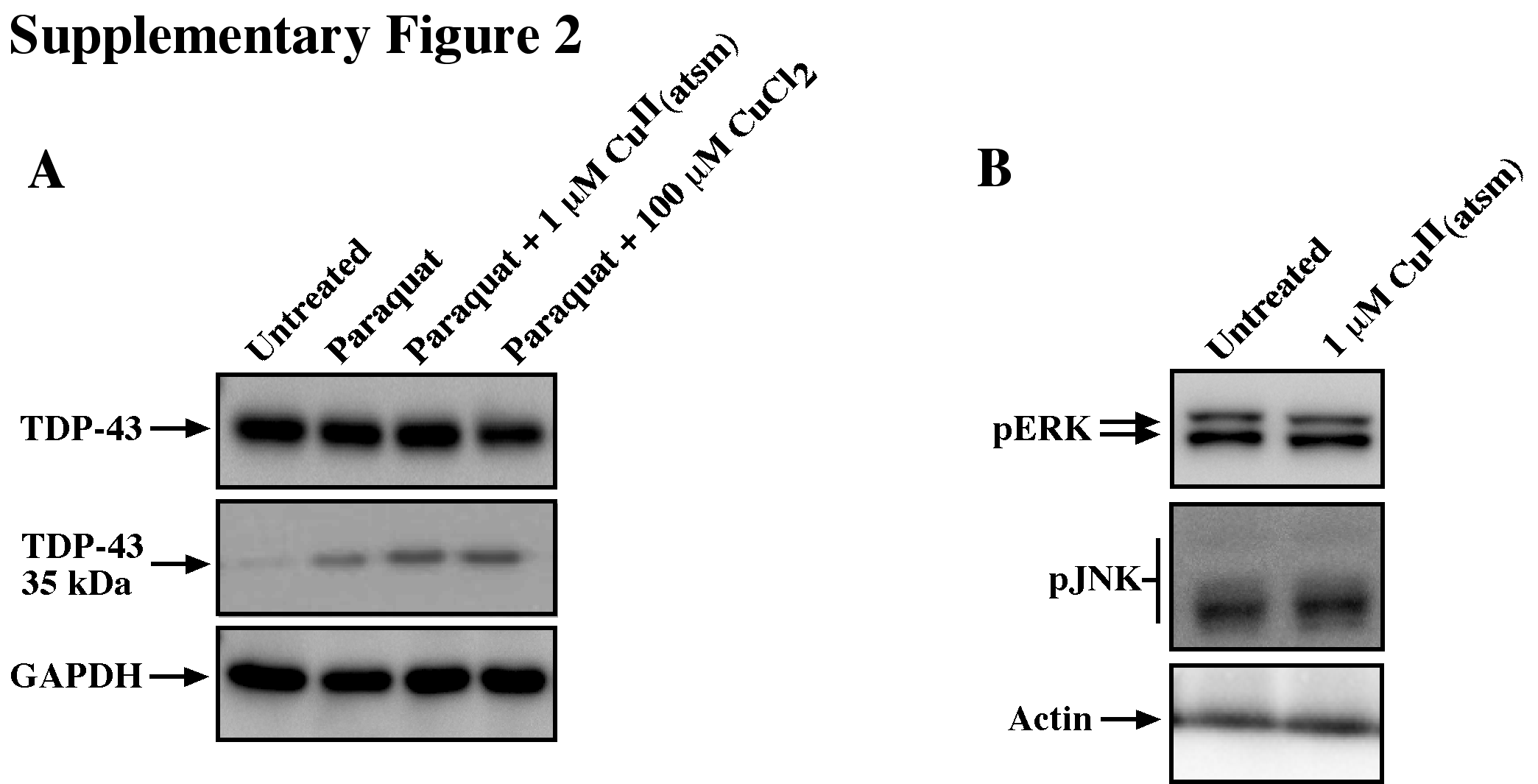

Supplement: Figure S2 — Effect of CuCl2 on TDP-43 expression. A: Cells were treated with 1 mM paraquat overnight in the presence or absence of 1 µM CuII(atsm) or 100 µM CuCl2. Cells were examined for expression of full length TDP-43 and 35 kDa CTF-TDP-43 by western blot. B: Effect of CuII(atsm) on ERK and JNK phosphorylation. Cells were treated overnight with 1 µM CuII(atsm) and examined for expression of phosphorylated ERK and JNK. (TIF) [file pone.0042277.s002.tif]
